# Plasma adiponectin levels and type 2 diabetes risk: a nested case-control study in a Chinese population and an updated meta-analysis

**DOI:** 10.1038/s41598-017-18709-9

**Published:** 2018-01-10

**Authors:** Yeli Wang, Rui-Wei Meng, Setor K. Kunutsor, Rajiv Chowdhury, Jian-Min Yuan, Woon-Puay Koh, An Pan

**Affiliations:** 1 Saw Swee Hock School of Public Health, National University of Singapore and National University Health System, Singapore, Singapore; 2 Department of Epidemiology and Biostatistics, Ministry of Education Key Laboratory of Environment and Health and State Key Laboratory of Environmental Health (incubation), School of Public Health, Tongji Medical College, Huazhong University of Science and Technology, Wuhan, Hubei Province China; 3 Translational Health Sciences, Bristol Medical School, University of Bristol Southmead Hospital, Bristol, United Kingdom; 4 Cardiovascular Epidemiology Unit, Department of Public Health and Primary Care, University of Cambridge, Cambridge, United Kingdom; 5 UPMC Hillman Cancer Center, University of Pittsburgh, Pittsburgh, Pennsylvania USA; 6 Department of Epidemiology, Graduate School of Public Health, University of Pittsburgh, Pittsburgh, Pennsylvania USA; 7 Duke-NUS Medical School, Singapore, Singapore

## Abstract

Results from previous prospective studies assessing the relation between adiponectin and type 2 diabetes (T2D) were not entirely consistent, and evidence in Chinese population is scarce. Moreover, the last meta-analysis did not examine the impact of metabolic variables on the adiponectin-T2D association. Therefore, we prospectively evaluated the adiponectin-T2D association among 571 T2D cases and 571 age-sex-matched controls nested within the Singapore Chinese Health Study (SCHS). Furthermore, we conducted an updated meta-analysis by searching prospective studies on Pubmed till September 2016. In the SCHS, the odds ratio of T2D, comparing the highest versus lowest tertile of adiponectin levels, was 0.30 (95% confidence interval: 0.17, 0.55) in the fully-adjusted model. The relation was stronger among heavier participants (body mass index ≥23 kg/m^2^) compared to their leaner counterparts (*P* for interaction = 0.041). In a meta-analysis of 34 prospective studies, the pooled relative risk was 0.53 (95% confidence interval: 0.47, 0.61) comparing the extreme tertiles of adiponectin with moderate heterogeneity (*I* ^2^ = 48.7%, *P* = 0.001). The adiponectin-T2D association remained unchanged after adjusting for inflammation and dyslipidemia markers, but substantially attenuated with adjustment for insulin sensitivity and/or glycaemia markers. Overall evidence indicates that higher adiponectin levels are associated with decreased T2D risk in Chinese and other populations.

## Introduction

Adiponectin, a major adipokine secreted by adipose tissue^1^, has attracted much attention due to its anti-inflammatory and insulin-sensitizing properties as well as its beneficial role in glucose metabolism^2^. Animal studies and experimental studies have shown that adiponectin improves insulin sensitivity^3,4^, thus may prevent the development of type 2 diabetes (T2D). A meta-analysis in year 2009 summarized data from 13 prospective studies in various populations, and confirmed an inverse association between adiponectin levels and T2D risk^5^, since then, many more studies have been published with variability in the strength of the adiponectin-T2D association^6–23^. Most of the previous studies have been conducted in the Western populations, and a few were conducted among Asian populations such as Japanese, Indians and Koreans. Chinese have lower adiponectin levels compared to other ethnic groups^24^. Meanwhile, Chinese are also particularly sensitive to the detrimental metabolic effects of greater body fatness^25^. So far only a relatively small study (76 T2D cases) has examined the association between adiponectin levels (binary variable) and T2D risk in a Hong Kong Chinese population in the context of T2D prediction model^26^. However, the shape of the association, and its potential confounders or modifying factors are unclear in a Chinese population.

The results from previous studies on the adiponectin-T2D relationship have not been entirely consistent. While some studies have reported a non-linear association^13,27^, others have observed a linear relationship^5,21^. Furthermore, data were conflicting on whether the association was modified by gender^10,13,15,17,28–31^ or levels of obesity^13,22,27,30,32^, and whether adiponectin improved T2D prediction significantly^6,16,26,33–37^. Moreover, the last meta-analysis pooled risk estimates from models that did not adjust for metabolic variables such as lipids, markers of inflammation, glycaemia and insulin sensitivity^5^.

In this context, we first conducted a case-control study nested within the prospective, population-based cohort, the Singapore Chinese Health Study (SCHS), to quantify the association between plasma adiponectin levels and risk of incident T2D in a Chinese population. We adjusted for blood lipids, inflammatory biomarker, glucose and insulin levels in the multivariable models to further test the potential mediation effect. We also assessed the incremental value of adiponectin in T2D risk prediction over established risk factors in this population. We further conducted an updated meta-analysis of published reports on circulating adiponectin and T2D, involving another 33 population-based longitudinal studies. We performed comprehensive stratified analysis to explore the heterogeneity among different subgroups and examined the dose-response relationship between adiponectin and T2D risk in the updated meta-analysis.

## Results

### Results in the SCHS

The process of selecting participants from SCHS in this study is presented in Supplemental Figure S1. The baseline characteristics of cases and controls are shown in Table 1. The mean age (standard deviation [SD]) of the participants was 59.7 (6.2) years and 58.7% were females. As expected, cases had high-risk profiles except for the matching factors, including higher body mass index (BMI) and higher prevalence of hypertension. No significant differences were found for education levels, smoking status, alcohol consumption and physical activity levels. For the plasma biomarkers, cases had higher levels of hemoglobin A1c (HbA1c), high-sensitivity C-reactive protein (hs-CRP), random glucose, random insulin, triglycerides (TG), and the ratio of TG to HDL-cholesterol (HDL-C), but lower HDL-C levels. The median (interquartile) concentration of adiponectin was 6.7 (5.2–8.3) µg/mL in men and 8.1 (6.4–10.5) µg/mL in women, and it was 6.7 (5.2–8.3) µg/mL in the cases and 8.4 (6.5–10.8) µg/mL in the controls.

**Table 1:** Baseline characteristics of cases and controls, case-control study nested within Singapore Chinese Health Study^a^.

	Cases (n = 571)	Controls (n = 571)	*P*-value^b^
Age (years) at blood taken	59.6 (6.1)	59.7 (6.2)	—
Gender (Female)	335 (58.7)	335 (58.7)	—
Dialect (%)			—
Cantonese	287 (50.3)	287 (50.3)	
Hokkien	284 (49.7)	284 (49.7)	
Body mass index, kg/m^2^	24.8 (3.6)	22.8 (3.3)	<0.001
Level of education (%)			0.15
No formal education	104 (18.2)	99 (17.3)	
Primary school	255 (44.7)	233 (40.8)	
Secondary and above	212 (37.1)	239 (41.9)	
History of Hypertension (%)	265 (46.4)	148 (25.9)	<0.001
Cigarette smoking (%)			0.08
Never smokers	410 (71.8)	425 (74.4)	
Former smoker	63 (11.0)	71 (12.4)	
Current smokers	98 (17.2)	75 (13.1)	
Weekly moderate-to-vigorous activity (%)			0.37
<0.5 hour/week	456 (79.9)	454 (79.5)	
0.5–3.9 hours/week	82 (14.4)	68 (11.9)	
≥4.0 hours/week	33 (5.8)	49 (8.6)	
Alcohol Intake (%)			0.89
Abstainers	498 (87.2)	497 (87.0)	
Weekly drinkers	55 (9.6)	59 (10.3)	
Daily drinkers	18 (3.2)	15 (2.6)	
Fasting status (yes)	178 (31.2)	156 (27.3)	0.15
Adiponectin, µg/mL	6.7 (5.2–8.3)	8.4 (6.5–10.8)	<0.001
High-sensitivity C-reactive protein, mg/L	1.8 (1.0–3.5)	1.2 (0.6–2.3)	<0.001
Random glucose, mmol/L	6.0 (4.8–8.9)	4.5 (4.1–5.3)	<0.001
Random insulin, mIU/L	14.7 (7.9–35.2)	9.0 (4.5–22.0)	<0.001
Total cholesterol, mmol/L	5.31 (0.95)	5.20 (0.85)	0.05
HDL cholesterol, mmol/L	1.08 (0.24)	1.23 (0.32)	<0.001
Triglycerides, mmol/L	2.2 (1.5–3.0)	1.5 (1.1–2.2)	<0.001
Ratio of triglycerides to HDL cholesterol	1.3 (0.8–2.1)	2.0 (1.3–3.0)	<0.001
Hemoglobin A1c, %	6.83 (1.44)	5.55 (0.27)	<0.001
Hemoglobin A1c, mmol/mol	51	38	<0.001

^a^Data are expressed as mean (SD) for continuous variables with normal distribution, median (interquartile range) for continuous variables with skewed distribution, and n (percentage) for categorical variables. Cases and controls are matched on age at blood taken (±3 years), gender, dialect, and date of blood collection ( ± 6 months).

^b^ *P*-values were based on conditional logistic regression.

Among the healthy control subjects, plasma adiponectin levels were inversely correlated with BMI, hs-CRP, random insulin, TG and the ratio of TG to HDL-C (Pearson’s coefficient *r* = −0.15, −0.19, −0.20, −0.43, and −0.50, respectively; all *P* < 0.001), and positively correlated with age and plasma HDL-C level (Pearson’s coefficient *r* = 0.11, and 0.49, *P* < 0.01) (Supplemental Table S1).

The association between adiponectin and risk of T2D is presented in Table 2. In the model with adjustment for T2D risk factors, higher plasma adiponectin levels were associated with a lower T2D risk, and the odds ratio (OR) and 95% confidence interval (CI) comparing the highest vs. lowest tertiles was 0.19 (0.12, 0.30; *P*-trend < 0.001). The association was slightly attenuated after adjusting for hs-CRP, and the ratio of TG to HDL-C (OR 0.31; 95% CI: 0.20, 0.51; *P*-trend < 0.001), and random glucose and insulin levels (OR 0.30; 95% CI: 0.17, 0.55; *P*-trend < 0.001). Moreover, significant associations were found in 292 case-control pairs with the cases having baseline HbA1c < 6.5% (48 mmol/mol) at the time of blood-taking, as well as in 279 case-control pairs with the cases having baseline HbA1c ≥6.5% (48 mmol/mol). In addition, the adiponectin-T2D association remained significant among 146 case-control pairs with HbA1c < 6.0% (42 mmol/mol). Significant interaction was found with BMI status (*P* for interaction = 0.041; Supplemental Table S2), and the association was stronger among overweight/obese subjects (BMI ≥23.0 kg/m^2^) than lean conterparts (BMI < 23.0 kg/m^2^). No statistically significant interactions were found with other variables.

**Table 2:** Odds ratios (95% confidence intervals) for risk of type 2 diabetes according to tertiles of adiponectin, the Singapore Chinese Health Study.

	Tertiles of adiponectin concentrations			*P* for trend^a^	per 1 log µg/mL
	T1	T2	T3		
***Whole dataset***					
Median (range)	5.5 (1.4, 7.1)	8.3 (7.2, 9.8)	11.8 (9.9, 29.9)		
Cases/controls	328/191	173/191	70/189		
Model 1^b^	1.00	0.55 (0.40, 0.76)	0.19 (0.12, 0.30)	<0.001	0.16 (0.10, 0.25)
Model 2^c^	1.00	0.67 (0.48, 0.95)	0.31 (0.20, 0.51)	<0.001	0.26 (0.16, 0.42)
Model 3^d^	1.00	0.80 (0.52, 1.23)	0.30 (0.17, 0.55)	<0.001	0.26 (0.14, 0.48)
***Limited to cases with baseline hemoglobin A1c*** ≥ ***6.5% (48 mmol/mol) and their matched controls***					
Median (range)	5.5 (1.4, 7.0)	8.1 (7.1, 9.7)	11.8 (9.8, 29.9)		
Cases/controls	159/98	88/92	32/89		
Model 1^b^	1.00	0.63 (0.41, 0.99)	0.19 (0.10, 0.37)	<0.001	0.13 (0.07, 0.25)
Model 2^c^	1.00	0.86 (0.53, 1.38)	0.35 (0.17, 0.71)	0.01	0.22 (0.11, 0.44)
Model 3^d^	1.00	2.08 (0.87, 4.98)	0.27 (0.08, 0.90)	0.20	0.26 (0.09, 0.82)
***Limited to cases with baseline hemoglobin A1c*** < ***6.5% (48 mmol/mol) and all the controls***					
Median (range)	5.7 (2.1, 7.5)	8.7 (7.6, 10.0)	12.1 (10.1, 26.7)		
Cases/controls	185/101	71/96	36/95		
Model 1^b^	1.00	0.38 (0.23, 0.62)	0.18 (0.09, 0.33)	<0.001	0.18 (0.10, 0.34)
Model 2^c^	1.00	0.47 (0.28, 0.80)	0.29 (0.15, 0.58)	<0.001	0.32 (0.16, 0.63)
Model 3^d^	1.00	0.57 (0.32, 1.04)	0.32 (0.14, 0.70)	0.003	0.32 (0.15, 0.70)
***Limited to cases with baseline hemoglobin A1c*** < ***6.0% (42 mmol/mol) and all the controls***					
Median (range)	5.9 (2.2, 7.5)	8.7 (7.6, 10.0)	12.2 (10.1, 26.7)		
Cases/controls	78/49	47/50	21/47		
Model 1^b^	1.00	0.56 (0.29, 1.09)	0.21 (0.09, 0.49)	<0.001	0.25 (0.11, 0.56)
Model 2^c^	1.00	0.79 (0.38, 1.63)	0.36 (0.14, 0.90)	0.037	0.44 (0.17, 1.10)
Model 3^d^	1.00	0.77 (0.34, 1.77)	0.34 (0.12, 0.98)	0.06	0.40 (0.14, 1.19)

^a^Linear trend was tested by using the median level of each tertile of adiponectin.

^b^Model 1: adjusted for age at blood taken (continuous), smoking (never, ever smoker), alcohol intake (never, ever drinker), weekly moderate-to-vigorous activity (<0.5, ≥0.5 hours/week), education level (primary school and below, secondary or above), history of hypertension (yes, no), fasting status (yes, no), and body mass index (continuous);

^c^Model 2: Model 1 plus adjusted for high-sensitivity C-reactive protein, and the ratio of triglycerides to high-density lipoprotein cholesterol (both in tertiles).

^d^Model 3: Model 2 plus adjusted for random glucose and random insulin (both in tertiles).

The summary statistics for the predictive performance of adiponectin is presented in the Supplemental Tables S3 and S4. The area under the receiver operating characteristic curve (AUC) for base model 1, which included education level, physical activity, history of hypertension, BMI, TG, HDL-C, and hs-CRP, was 0.74 (95% CI: 0.71, 0.77). Addition of adiponectin showed statistically significant improvement in AUC (AUC change = 0.02, *P* < 0.05), reclassified 33% individuals more appropriately (net reclassification improvement [NRI] = 0.33, *P* < 0.001) and led to an integrated discrimination improvement (IDI) of 0.03 (*P* < 0.001). Base model 2, with additional inclusion of random glucose and insulin levels, had an AUC of 0.82 (95% CI: 0.80, 0.85). Adding adiponectin did not improve AUC significantly, but resulted in statistical significant NRI (0.19, *P* = 0.003) and IDI (0.01, *P* < 0.001), indicating that the difference in average predicted risks between individuals with and without T2D increased significantly when adiponectin was included in the prediction model. Base model 3, substituting random glucose in base model 2 with HbA1c, had an AUC of 0.87 (95% CI: 0.84, 0.89), which was significantly higher than the AUC of base model 2 (AUC change = 0.05, *P* < 0.001). Similar to base model 2, adding adiponectin to base model 3 did not improve AUC, but resulted in small yet marginally significant NRI (0.12, *P* = 0.051) and IDI (0.01, *P* < 0.05).

### Meta-analysis

Our initial search identified 2159 potentially relevant citations (Supplemental Figure S2). After screening for title, abstract and full texts, we included 33 prospective cohort studies based on 31 articles (2 articles reported results for 4 cohort studies), and the characteristics of the studies are shown in the Tables 3–4. Eighteen studies included participants from Europe, 9 studies from North America, and 6 studies from Asia. There were 7504 T2D cases among 64,696 participants including our study. The duration of follow-ups varied from 1 to 18 years, the mean ages ranged from 26.5 to 74.8 years. Four studies were conducted in men only^19,23,32,38^, and two studies were in women only^9,27^, while 28 studies were in both sexes, among which five studies provided risk estimates for men and women separately^7,10,15,17,28^. Furthermore, study qualities ranged from 5 to 9 (Table 3).

**Table 3:** Baseline characteristics of the 33 prospective studies included in the meta-analysis

Reference, Year	Study name or source of participants	Study location	Study design	Follow-up years	Mean baseline age or range	Female (%)	Number of cases/non-cases	Adiponectin assay	Ascertainment of type 2 diabetes	Study quality
Lindsay *et al*.^57^	Gila River Indian Community	USA	Nested case-control	6.7	32	66	70/70	ELISA	OGTT	7
Daimon *et al*.^58^	Funagata study	Japan	Cohort	5	58	58	18/819	ELISA	OGTT	7
Snehalatha *et al*.^59^	Indian Diabetes Prevention Program	India	Cohort	1	45	42	25/66	RIA	OGTT	5
Choi *et al*.^60^	South-West Seoul	Korea	Cohort	3	70	NA	25/294	RIA	OGTT	6
Kanaya *et al*.^61^	Health ABC	USA	Cohort	5	74	53	143/2213	RIA	(1) self-report; (2) medication intake; (3) OGTT	8
Koenig *et al*.^38^	MONICA Ausburg	Germany	Cohort	18	54	0	115/772	ELISA	confirmed self-report	6
Nakashima *et al*.^62^	Hawaii-Los Angeles-Hiroshima Study	USA	Cohort	5.4	61.3	58	112/654	ELISA	OGTT	7
Snijder *et al*.^28^	The Hoorn Study	Netherlands	Cohort	6.4	60	54	118/1146	Latex turbidimetric immunoassay	OGTT	8
Wannamethee *et al*.^32^	British Regional Heart Study	UK	Cohort	5	69	0	105/3462	ELISA	Confirmed self-report	7
Heidermann *et al*.^27^	Nurses’ Health Study	USA	Nested case-control	12	56	100	1038/1136	ELISA	Confirmed self-report	7
Ley *et al*.^63^	Sandy Lake Health and Diabetes Project cohort	Canada	Cohort	10	27	58	86/406	RIA	(1) self-report; (2) medication intake; (3) OGTT	8
Mather *et al*.^31^	Diabetes Prevention Program	USA	Cohort	1	51	68	115/925	Latex turbidimetric immunoassay	OGTT	5
Tábak *et al*.^64^	Whitehall II Study	UK	Nested case-control	11.5	51	31	55/85	Bio-Plex Suspension assay	(1) self-report; (2) medication intake; (3) OGTT	8
Salomaa *et al*.^6^	FINRISK97	Finland	Cohort	10.8	46	50	417/7410	ELISA	(1) medication; (2) hospital record and death registry	8
	Health 2000 cohort	Finland	Cohort	7.1	53	54	179/4798	ELISA	(1) medication; (2) hospital record and death registry	8
Thorand *et al*.^7^	MONICA/KORA	Germany	Case-cohort	10.9	53	49	460/1474	ELISA	Confirmed self-report	8
Zhu *et al*.^8^	ARIC	USA	Case-cohort	9	45–64	63	550/540	ELISA	(1) Physician diagnosis; (2) medication use; (3) OGTT	9
Fagerberg *et al*.^9^	population-based cohort of 64-year-old women	Sweden	Cohort	5.5	70	100	69/272	ELISA	OGTT	9
Hanley *et al*.^10^	IRAS Family Study	USA	Cohort	5	41	61	82/1014	RIA	(1) OGTT; (2) medication use	8
Hivert *et al*.^11^	KORA S4/F4	Germany	Cohort	8	63	49	93/794	RIA	(1) Physician diagnosis; (2) OGTT	8
	Framingham Offspring Study	USA	Cohort	6.5	60	56	109/1914	ELISA	(1) OGTT; (2) medication use	8
Montonen *et al*.^12^	EPIC-Potsdam	Germany	Case-cohort	7	51	58	613/1965	ELISA	Confirmed self-report	7
Kizer *et al*.^13^	Cardiovascular Health Study	USA	Cohort	10.6	75	63	309/3493	ELISA	(1) use of medication; (2) OGTT	8
Li *et al*.^14^	local government workers from Aichi perfecture	Japan	Cohort	5.3	47	23	164/2844	ELISA	(1) OGTT; (2) self-report	9
Lilja *et al*.^15^	Västerbotten Intervention Program	Sweden	Case-referent	17	53	48	640/1564	double antibody RIAs	OGTT	9
Marques-Vidal *et al*.^16^	The CoLaus Study	Switzerland	Cohort	5.5	52	57	208/3634	ELISA	(1) OGTT; (2) presence of oral hypoglycaemic; (3) insulin treatment	9
Kim *et al*.^17^	Seuol Metabolic Syndrome Research Initiatives	Korea	Cohort	4.4	46	20	652/4433	ELISA	(1) OGTT; (2) 3 outpatient treatment; (3) hospitalization due to type 2 diabetes	7
Rubio-Martin *et al*.^18^	Pizarra cohort study	Spain	Cohort	5	44	65	52/417	EIA	OGTT	8
Sans *et al*.^19^	MONICA-Catalonia	Spain	Cohort	9.4	50	0	85/799	Luminex xMAP technology	(1) OGTT; (2) self-report	8
Julia *et al*.^20^	SU.VI.MAX study	France	Nested case-control	13	51	51	82/1263	ELISA	(1) OGTT; (2) medication	9
Lindberg *et al*.^21^	Patients with Myocardial infarction	Denmark	Cohort	5.7	64	26	38/628	Immunoturbidimetric assay	Registry, validated using medical records	7
Yamamoto *et al*.^22^	Hitachi Health Study	Japan	Cohort	3	52	10	214/4377	Immunoturbidimetric assay	(1) OGTT; (2) HbA1c ≥6.5%; (3) under diabetes treatment	9
Neville *et al*.^23^	PRIME study	Ireland	Cohort	14.7	55	0	151/1688	ELISA	Confirmed self-report	8

Abbreviations: Health ABC, Health, Aging and Body Composition; MONICA, MONIotring of trends and determinants in CArdiovascular disease; KORA, Cooperative Health Research in the Region of Augsburg; ARIC, The Atherosclerosis Risk in Communities Study; IRAS, The insulin resistance atherosclerosis study; EPIC, The European Prospective Investigation into Cancer and Nutrition study; SU.VI.MAX, SUpplementation en VItamines et Mineraux AntioXydants; PRIME, The Prospective Epidemiological Study of Myocardial Infarction; RIA, radioimmunoassays; EIA, enzyme immunoassay.

**Table 4:** Results of the 33 prospective studies included in the meta-analysis.

Reference, Year	Comparison	Model	RR 95% CI	Adjustment for covariates
Lindsay *et al*.^57^	Per SD increment	Multivariable	0.59 0.38, 0.91	Age, waist circumference, fasting and 2-h glucose, fasting insulin. Matched for BMI, age and sex
Daimon *et al*.^58^	Highest tertile (median, 13.9 μg/mL) vs lowest (4.7 μg/mL)	Multivariable	0.11 0.01, 0.96	Age, sex, waist-hip ratio, 2-h glucose, TNF-_1_
Snehalatha *et al*.^59^	Per 1 μg/mL increment	Multivariable	0.87 0.79, 0.95	HbA1c
Choi *et al*.^60^	Highest tertile (median, 23.8 μg/mL) vs lowest (7.9 μg/mL)	Multivariable	0.31 0.14, 0.71	BMI
Kanaya *et al*.^61^	per 1 log μg/mL	Multivariable	1.04 0.69, 1.56	Age, sex, race, BMI, visceral fat, hypertension, leptin, PAI-1, fasting glucose and insulin, HDL-C, TG
Koenig *et al*.^38^	Highest tertile (median, 10.6 μg/mL) vs lowest (3.8 μg/mL)	Multivariable	0.55 0.35, 0.89	Age, BMI, smoking, alcohol intake, physical activity, hypertension, history of myocardial infarction
		Model 2	0.81 0.50, 1.33	Additional adjustment for HDL-C
Nakashima *et al*.^62^	Highest tertile (median, 17.4 μg/mL) vs lowest (5.4 μg/mL)	Multivariable	0.56 0.32, 0.99	Age, sex, BMI, waist-hip ratio, HOMA-IR, glucose tolerance classification
Snijder *et al*.^28^	Highest quartile (median, 28.4 μg/mL in men, 24.8 μg/mL in women) vs lowest (8.1 μg/mL in men, 8.5 μg/mL in women)	Multivariable	0.43 0.19, 0.94	Age, waist-hip ratio, smoking, performance of sports, leptin
		Model 2	0.72 0.36, 1.42	Additional adjustment for fasting and 2-h glucose
		Model 3	0.75 0.38, 1.51	Additional adjustment for TG
Wannamethee *et al*.^32^	Highest tertile (median, 13.7 μg/mL) vs lowest (3.6 μg/mL)	Multivariable	0.40 0.23, 0.70	Age, BMI, social class, physical activity, smoking, alcohol intake, history of coronary heart disease or stroke, statin use, blood pressure, treatment for hypertension
		Model 2	0.59 0.33, 1.04	Additional adjustment for HOMA-IR
		Model 3	0.67 0.38, 1.20	Additional adjustment for HDL-C and CRP
Heidermann *et al*.^27^	Highest quintile (median, 28.4 μg/mL) vs lowest (8.1 μg/mL)	Multivariable	0.17 0.12, 0.25	Age, BMI, ethnicity, physical activity, smoking, family history of diabetes, hormone therapy, alcohol intake, dietary factors. Matched for age at blood draw ( ± 1 year), date of blood draw ( ± 3 months), fasting status, race
		Model 2	0.16 0.10, 0.27	Additional adjustment for hyperlipidemia, hypertension and CRP
		Model 3	0.26 0.13, 0.51	Additional adjustment for fasting insulin
Ley *et al*.^63^	Per SD increment	Multivariable	0.68 0.51, 0.90	age, sex, waist circumference, TG, HDL-C, hypertension, IGT
Mather *et al*.^31^	Per SD increment	Multivariate	0.77 0.66, 0.89	age, sex, race/ethnicity
		Model 2	0.84 0.71, 0.98	Additional adjustment for change in weight, change in adiponectin and baseline and change in insulinogenic index, 1/fasting insulin
Tábak *et al*.^64^	Per 1 μg/mL increment	Multivariable	0.87 0.77, 0.97	Age, sex, BMI, physical activity, family history of diabetes, employment grade. Matched for matched on sex, age (5-year groups), and BMI (5 kg/m^2^ groups)
		Model 2	0.89 0.79, 0.99	Age, sex, BMI, TC, TG, blood pressure
		Model 3	0.89 0.79, 1.00	Additional adjustment for CRP
		Model 4	0.94 0.82, 1.07	Additional adjustment for fasting glucose
Salomaa *et al*.^6^	Per SD increment	Multivariable	0.67 0.61, 0.80	Sex, non-HDL-C, HDL-C, TG, BMI, systolic blood pressure, current smoking, blood glucose, history of cardiovascular disease event, use of antihypertensive medication
	Per SD increment	Multivariable	0.70 0.60, 0.90	Sex, non-HDL-C, HDL-C, TG, BMI, systolic blood pressure, current smoking, blood glucose, history of cardiovascular disease event, use of antihypertensive medication
Thorand *et al*.^7^	Highest tertile (median 13.3 μg/mL in men, 18.1 μg/mL in women) vs. lowest tertile (6.7 μg/mL in men, 9.9 μg/mL in women)	Multivariable	0.28 0.20, 0.39	Age, sex, survey, BMI, smoking, alcohol consumption, physical activity
		Model 2	0.38 0.27, 0.53	Additional adjustment for systolic blood pressure, TC/HDL-C, parental history of diabetes mellitus, CRP, interleukin-6, soluble ICAM-1 and soluble E-selection, and leptin
Zhu *et al*.^8^	Highest quartile (weighted median: 10.61 μg/mL) vs. lowest (weighted median: 3.48 μg/mL)	Multivariable	0.40 0.25, 0.64	Age, sex, ethnicity, center, hypertension, and parental history of diabetes, BMI, waist-to-hip ratio
		Model 2	0.46 0.29, 0.74	Additional adjustment for inflammation score
		Model 3	0.52 0.32, 0.85	Additional adjustment for fasting insulin
		Model 4	0.82 0.48, 1.42	Additional adjustment for fasting glucose
Fagerberg *et al*.^9^	Highest tertile (18.28–40.78 μg/mL) vs lowest tertile (3.68–11.36 μg/mL)	Multivariable	0.22 0.07, 0.69	HOMA-IR, AIR, smoking, IFG, IGT
Hanley *et al*.^10^	per SD increment	Multivariable	0.67 0.46, 0.97	Age, sex, ethnicity, smoking, BMI
		Model 2	0.64 0.43, 0.94	Age, sex, ethnicity, smoking, HDL-C
		Model 3	0.69 0.49, 0.99	Age, sex, ethnicity, smoking, HOMA-IR
		Model 4	0.81 0.56, 1.16	Age, sex, ethnicity, smoking, S_1_
		Model 5	0.75 0.53, 1.06	Age, sex, ethnicity, smoking, IFG
Hivert *et al*.^11^	per SD increment	Multivariable	0.57 0.40, 0.81	Age, sex, BMI
		Model 2	0.58 0.41, 0.84	Additional adjustment for HOMA-IR
	per SD increment	Multivariable	0.53 0.39, 0.74	Age, sex, BMI
		Model 2	0.69 0.51, 0.96	Additional adjustment for HOMA-IR, resistin, TNF-alpha
Montonen *et al*.^12^	Highest quintile (median 9.7 μg/mL in men, 14.4 μg/mL in women) vs. lowest quintile (3.07 μg/mL in men, 4.74 μg/mL in women)	Multivariable	0.18 0.12, 0.28	Age, sex, education, sport activity, cycling, occupational activity, smoking, alcohol intake, consumptions of red meat, whole grain bread and coffee, BMI, waist-circumference
		Model 2	0.26 0.16, 0.40	Additional adjustment for GGT, HDL-C, hs-CRP
		Model 3	0.28 0.17, 0.44	Additional adjustment for HbA1c
Kizer *et al*.^13^	Highest quartile (median 23.6 μg/mL) vs. lowest (median 7.2 μg/mL)	Multivariable	0.41 0.28, 0.61	Age, sex, race, income, smoking, alcohol, eGFR, prevalent congestive heart failure, prevalent atrial fibrillation, prevalent CHD, beta-blocker use, health status, BMI
		Model 2	0.79 0.50, 1.23	Additional adjustment for systolic blood pressure, HDL-C, TG, CRP, HOMA-IR
Li *et al*.^14^	Highest quintile (median 13.9 μg/mL) vs. lowest quintile (median 4.3 μg/mL)	Multivariable	0.72 0.42, 1.25	Age, sex, smoking, physical activity, alcohol consumption, family history of diabetes, BMI
		Model 2	0.85 0.48, 1.49	Additional adjustment for CRP, fasting blood glucose, insulin
Lilja *et al*.^15^	Highest quartiles (≥12.1 μg/mL in men, ≥18.4 μg/mL in women) vs. lowest quartile (≤6.2 μg/mL in men, ≤9.2 μg/mL in women)	Multivariable	0.40 0.30, 0.54	BMI
		Model 2	0.49 0.35, 0.70	Additional adjustment for TC, hypertension, smoking, physical activity, university education, first-degree diabetes heredity, fasting and postload glucose
		Model 3	0.55 0.38, 0.78	Additional adjustment for HOMA-IR
Marques-Vidal *et al*.^16^	Highest quartile (mean 17.3 μg/mL) vs. lowest quartile (mean 3.7 μg/mL)	Multivariable	0.41 0.26, 0.65	Age, gender, BMI
		Model 2	0.64 0.40, 1.03	Additional adjustment for diabetes risk score (age, family history of type 2 diabetes, height, waist circumference, resting heart rate, presence of hypertension, HDL-C, TG, fasting glucose and serum uric acid)
Kim *et al*.^17^	Highest tertil (≥6.23 μg/mL in men, ≥9.47 μg/mL in women) vs. lowest tertile (<3.90 μg/mL in men, <6.01 μg/mL in women)	Multivariable	0.67 0.46, 0.96	Age, sex, BMI, waist circumferences
		Model 2	0.67 0.45, 1.00	Additional adjustment for fasting serum glucose
Rubio-Martin *et al*.^18^	Highest tertile (>13.2 μg/mL) vs. lowest tertile (<6.6 μg/mL)	Multivariable	0.24 0.07, 0.82	Age, sex, obesity, CRP
Sans *et al*.^19^	Per 1 log increase	Multivariable	0.22 0.08, 0.61	Age, BMI, leptin
		Model 2	0.25 0.09, 0.70	Age, BMI, insulin, years of school
		Model 3	0.24 0.08, 0.72	Age, BMI, leptin, years of school, DBP, HDL-C, TG
		Model 4	0.47 0.16, 1.40	Age, BMI, leptin, fasting glucose, years of school, DBP, HDL-C, TG
Julia *et al*.^20^	Highest tertile vs. lowest tertile	Multivariable	0.56 0.27, 1.18	Age, sex, supplementation group, family history of diabetes and BMI. Matched for sex, age, BMI and initial supplementation group.
		Model 2	0.71 0.33, 1.53	Additional adjustment for baseline glycaemia, TC and TG
Lindberg *et al*.^21^	Highest quartile (>10.35 μg/mL) vs. lowest quartile (≤5.13 μg/mL)	Multivariable	0.16 0.04, 0.66	Age and sex
		Model 2	0.17 0.04, 0.75	Additional adjustment for hypertension, hypercholesterolemia, current smoking, previous MI, BMI, blood glucose
		Model 3	0.15 0.02, 0.90	Additional adjustment for TC, HDL-C, LDL-C, TG
Yamamoto *et al*.^22^	Highest quartile (≥9.6 μg/mL) vs. lowest (<5.2 μg/mL)	Multivariable	0.40 0.25, 0.64	Age, sex, family history, smoking, alcohol drinking, physical activity, BMI
		Model 2	0.53 0.33, 0.86	Additional adjustment for HOMA-IR
		Model 3	0.56 0.35, 0.91	Age, sex, family history, smoking, alcohol drinking, physical activity, BMI, HbA1c
		Model 4	0.69 0.42, 1.13	Additional adjustment for HOMA-IR
Neville *et al*.^23^	Highest tertile (>6.66 μg/mL) vs. lowest (<3.77 μg/mL)	Multivariable	0.29 0.17, 0.52	Age, BMI, waist/hip ratio, alcohol status, smoking status, measures of socioeconomic status (includes material conditions and deprivation score), physical activity
		Model 2	0.37 0.20, 0.67	Additional adjustment for TC, HDL-C, TG, systolic blood pressure, on drug treatment for hypertension, CRP
		Model 3	0.54 0.29, 0.99	Additional adjustment for HOMA-IR

Abbreviations: OR, odds ratio; CI, confidence interval; TNF-_1_, the tumour necrosis factor 1; PAI-1, plasminogen activator inhibitor 1; TC, total cholesterol; LDL-C, LDL cholesterol; HDL-C, HDL cholesterol; TG, triglycerides; TG/HDL-C, the ratio of TG to HDL-C; CRP, C-reactive protein; hs-CRP, high-sensitivity CRP; IGT, impaired glucose tolerance; IFG, impaired fasting glucose; GGT, gamma-glutamyltransferase; ICAM-1, intercellular adhesion molecule 1; S_1_, insulin sensitivity index; eGFR, the epidermal growth factor receptor; CHD, coronary heart disease; DBP, diastolic blood pressure; MI, myocardial infarction.

**Figure 1:**
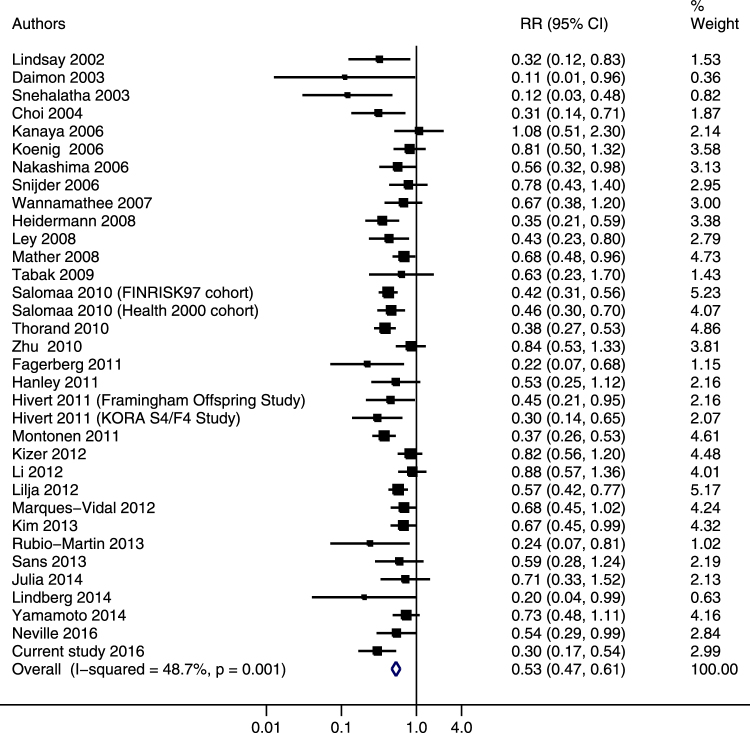
Adjusted relative risks of adiponectin levels with risk of type 2 diabetes in the updated meta-analysis. The summary estimates were obtained from the most fully-adjusted models of each study using a random-effects model. The data markers indicate the adjusted relative risks (RRs) comparing extreme tertiles of adiponectin levels. The size of the data markers indicates the weight of the study, which is the inverse variance of the effect estimate. The diamond data markers indicate the pooled RRs.

The pooled relative risk (RR) comparing the highest to lowest tertile of adiponectin concentrations was 0.53 (95% CI: 0.47, 0.61) using the random-effects model (Figure 1), and results were similar when using the fixed-effects model (RR 0.54; 95% CI: 0.50, 0.59). A moderate heterogeneity was observed (*I* ^2^ = 48.7%; *P* = 0.001), and the meta-regression analysis suggested evidence of effect modification by level of adjustment (*P* = 0.08) (data not shown). We further performed stratified analysis by level of adjustment, and the association was attenuated after further adjustment for glycaemia markers and/or insulin sensitivity markers, while additional adjustment for lipids and/or inflammatory markers did not materially change the association (Supplemental Table S5). When stratifying by baseline participant characteristics, the inverse association was consistently observed among all subgroups (Supplemental Table S5).

**Figure 2:**
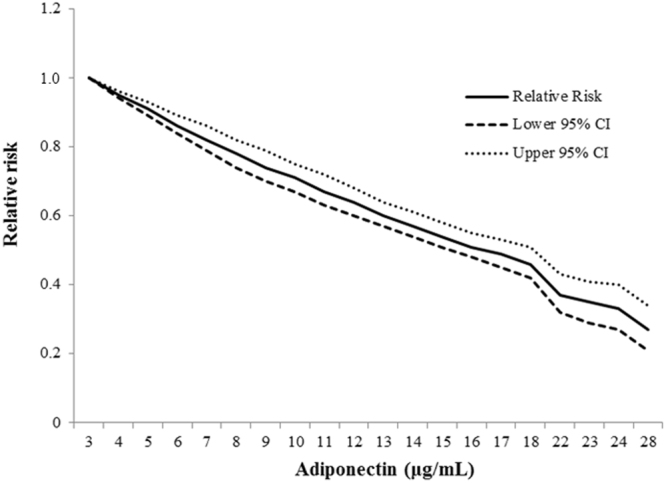
The association between concentrations of adiponectin and type 2 diabetes in the updated meta-analysis. The *Solid line* represents point estimates of relative risk for the adiponectin-diabetes association, and the *dotted lines* represent the upper and lower bound of 95% CIs. Cubic spline analysis was used to examine the association between adiponectin concentrations (categorical) and risk of developing type 2 diabetes using the most fully-adjusted models from reported studies. *P* = 0.33 for nonlinearity in the cubic spline regression model.

Twelve studies (14 data points because two studies reported results for men and women separately^10,28^) provided data for dose-response meta-analysis (Supplemental Table S6). Cubic spline regression model suggested a linear dose-response relationship (*P* = 0.33 for nonlinearity) (Figure 2). The RRs of T2D with per 5 µg/mL increment in adiponectin levels were 0.75 (95% CI: 0.73, 0.77) in the random-effects model. No significant publication bias was detected (*P* = 0.12 in the Egger’s test).

## Discussion

In this nested case-control study, we found a strong dose-dependent association between higher plasma adiponectin levels and lower T2D risk, which seemed to be independent of established T2D risk factors, including lipids, inflammatory biomarker (hs-CRP), random glucose and insulin. Moreover, the inverse association was significantly stronger among overweight/obese subjects than their lean counterparts. Furthermore, adiponectin significantly improved T2D risk reclassification in this population. In an updated meta-analysis which pooled data from 34 prospective studies including the current study, participants in the highest tertile had almost half the risk of developing T2D compared to those in the lowest tertile (RR 0.53; 95% CI: 0.47, 0.61), and the adiponectin-T2D association appeared to be linear.

A previous meta-analysis of 13 prospective studies (2623 incident T2D cases and 14,598 participants) reported an inverse association between adiponectin levels and T2D risk (RR 0.72 for per 1-log µg/mL increment in adiponectin levels) across different populations^5^, but no study was done in Chinese population. In the current study, we not only observed the inverse association in a Chinese population after comprehensive adjustment, but also conducted an updated meta-analysis with a much larger sample size (7504 incident T2D cases and 64,696 participants), which provided strong evidence for the role of adiponectin in the pathogenesis of T2D across diverse populations, a consistent finding despite methodologic variations such as adiponectin assays, diagnostic criteria for T2D and follow-up durations.

Several lines of evidence have hypothesized that adiponectin may act against T2D development by lowering blood glucose through improving insulin sensitivity. Mechanistic studies have shown that adiponectin improves insulin sensitivity by stimulating glucose utilization and fatty acid oxidation in the skeletal muscle and liver through improving AMP-activated protein kinase^4^. Animal studies have shown that adiponectin levels drop in parallel with reduced insulin sensitivity, before the increment in glycemic levels^3^. A recent Mendelian randomization study in humans provided further evidence between increased adiponectin levels and improved insulin sensitivity using ADIPOQ SNPs as instruments^39^. Additionally, we found in our current meta-analysis that the adiponectin-T2D association was substantially attenuated in studies that adjusted for insulin sensitivity and/or glycaemia markers compared to studies that did not adjust for either one of them, suggesting that the association was at least partially mediated through insulin and glucose pathways. Moreover, the stratified analysis from the current meta-analysis suggested that lower adiponectin levels may contribute to an increased T2D risk through pathways independent of other important T2D pathogenic mechanisms such as dyslipidemia and inflammation. Although adiponectin is an adipose-specific protein and its concentration reduces with increasing level of obesity^40^, the current cohort study together with previous studies^10,11,22^ have shown that adjustment for markers of obesity, such as BMI or CT-assessed abdominal fat area, did not materially change the association between adiponectin and T2D risk, suggesting that low adiponectin levels could be associated with higher T2D risk through pathways that may not be related to systemic or regional fat deposition.

Moreover, our study found stronger relation of adiponectin with T2D risk in overweight/obese people than their leaner counterparts. In line with our results, a 4-year follow-up study of elderly British men found stronger association among obese participants (BMI ≥30.0 kg/m^2^) than non-obese counterparts^32^. Similarly, a Japanese cohort study reported stronger association in overweight/obese individuals (BMI ≥25.0 kg/m^2^) than in normal weight people^22^. Some other studies also found that the association between adiponectin levels and T2D were stronger in participants with higher metabolic risk profile^11,17,41^. A recent Mendelian randomization study in a Swedish population also observed a stronger causal relation between adiponectin and insulin sensitivity in participants with higher BMI^39^. Although the underlying mechanism for the observed interaction with obesity is still unclear, the Mendelian randomization study hypothesized that the observed interaction with BMI may be due to the interaction between adiposity and the ADIPOQ SNPs, in which the transcription factors could have been regulated by the inflammatory mediators to stimulate the binding site and thus cause up-regulation of the related gene^39^. However, given the multiple statistical tests for interaction conducted and that some studies did not observe significant interactions with BMI^13,27,30^, our observed interaction with BMI should be interpreted with caution.

In the current study, adding adiponectin beyond traditional risk factors and glucose markers (random glucose or HbA1c) did not improve discrimination (assessed by AUC), but improved risk reclassification (assessed by NRI and IDI) for T2D. In consistent with our results, two cohort studies from Switzerland and Germany did not observe significant improvement in AUC when adding adiponectin alone to base models that included fasting glucose^16,34^. In contrast, results from a cohort in Hong Kong and EPIC-Potsdam study have shown that adiponectin improved AUC significantly beyond glucose markers^26,37^, and interestingly, in a previous study conducted in Finland, adiponectin improved AUC and NRI significantly in the primary cohort (FINRISK97) but not in the validation cohort (Health 2000)^6^. In addition, a T2D score comprising adiponectin and other five biomarkers has shown better predictive utility (assessed by AUC) than HbA1c and fasting glucose in a Danish population^33^, and the predictive utility of the score was further validated in two cohorts from Finland^35^ and US^36^ using AUC and NRI.

Our study has several strengths. First, we have adjusted for well-established T2D factors and used comprehensive statistical methods to explore the predictive utility of adiponectin. Moreover, compared to the previous meta-analysis^5^, our updated meta-analysis included more prospective studies with a tripled sample size; we have also included results from models that have adjusted for a comprehensive panel of T2D biomarkers (lipids and markers of inflammation, glycaemia and insulin sensitivity) to examine their impact on the adiponectin-T2D association, and further performed stratified analysis by these factors to explore potential heterogeneity among subgroups. Furthermore, we have performed a dose-response meta-analysis among 12 studies, and observed a linear association between adiponectin and T2D. However, the current study has some limitations as well. First, we measured adiponectin levels only once and some measurement errors are inevitable. In addition, the height and weight were self-reported and residual confounding was possible. Hence, this could lead to non-differential misclassifications and result in an underestimation of the true effect size of adiponectin on T2D risk. Furthermore, information of more precise surrogates for insulin resistance (such as fasting insulin, HOMA-IR, and insulin sensitivity index) and glycaemia markers (such as fasting glucose) were not available in the study, thus, random insulin and glucose levels were used as covariates in our models. Moreover, incident T2D was obtained from self-reported information, thus undiagnosed T2D may exist. However, we have used HbA1c as a selection criterion (<6.0%) for controls to minimize bias due to undiagnosed T2D. In addition, we also conducted sensitivity analysis restricting to cases with HbA1c <6.5% and <6.0% at the time of blood collection and their respective controls, and observed similar adiponectin-T2D association. Furthermore, the present study was conducted in a middle-aged and elderly population with a higher T2D incidence and adiponectin levels, and the findings may not be applicable to younger age group^28^. However, in the current meta-analysis, we did not observe any heterogeneity of results by age group, suggesting the finding will be most likely to be observed in younger people as well.

## Materials and Methods

### Study population

The design of the SCHS was described in detail previously^42^. Briefly, the SCHS was established between 1993 and 1998, and recruited 63,257 Chinese adults aged 45–74 years. At recruitment, an in-person interview was conducted using a structured questionnaire to collect information of diet, lifestyle habits and medical history. Follow-up I interviews were conducted via telephone between 1999 and 2004 to update selected lifestyle habit and medical history. A total of 52,322 participants were re-contacted successfully, and among them, a total of 32,535 participants donated their bio-specimens during follow-up I visits. Follow-up II interviews were conducted via telephone from 2006 to 2010, and 39,528 participants were re-contacted successfully. Among the 32,535 participants who donated bio-specimens, 25,477 (78.3%) were re-contacted for follow-up II interviews. The study protocol was approved by the Institutional Review Boards at the National University of Singapore and the University of Pittsburgh, and all methods were performed in accordance with the relevant guidelines and regulations. Informed consent was provided with completion of the baseline interview.

### Ascertainment of diabetes and other covariates

History of physician-diagnosed diabetes was asked at baseline and both follow-up interviews using the question: “Have you been told by a doctor that you have diabetes?” If the answer was “yes”, participants were also asked for the age at which they were first diagnosed. The robustness and accuracy of the self-reported diabetes data has been confirmed in a validation study: among 1651 cohort participants who reported history of diabetes either at baseline or follow-up I interview, 98.9% were confirmed by medical records or telephone interview. Some participants (n = 619) refused or were not available for the validation study, but their main characteristics (age, sex, BMI etc.) were similar to those in the validation study^43^.

Body weight and height were self-reported at baseline and both follow-ups. BMI is calculated as weight (kilograms) divided by height (meters) squared. For those with missing weight or height, BMI was calculated using imputed weight or height derived from the linear regression equation: Weight = y-intercept + gradient × height, where values for the y-intercept and gradient were derived from gender-specific weight-height regression lines obtained from all cohort participants with known heights and weights.

### Establishment of nested case-control study

For the current analysis, we established a nested case-control study of 571 cases and 571 matched controls within this cohort. All cases and controls were free of physician-diagnosed T2D, cardiovascular disease and cancer at baseline interview as well as the time of blood collection during 1999 and 2004. Cases were a total of 571 participants who subsequently reported to be diagnosed with incident T2D during follow-up II visit (2006–2010). Controls were chosen from the remaining participants who did not develop T2D or cardiovascular disease at follow-up II, and were matched for age (±3 years), date (±6 months) of blood collection, sex and dialect group with the cases on a 1:1 ratio. Furthermore, the selected controls were screened for the presence of undiagnosed T2D at the time of blood donation by HbA1c measurements. All matched controls with HbA1c ≥6.0% (42 mmol/mol) were considered ineligible for the study and a replacement control with the same matching criteria was randomly chosen among the remaining eligible subjects.

### Laboratory procedures

Random morning peripheral blood samples were obtained, and frozen plasma aliquots from the cases and controls were analyzed simultaneously in the same batch at the National University Hospital Reference Laboratory, Singapore. Adiponectin levels were measured by ELISA/Evolis (Bio-Rad Laboratories, Hercules, CA). The within-assay and between-assay coefficients of variation were 3.9–5.9% and 6.3–7.0%, respectively. Plasma hs-CRP levels and blood lipids (total cholesterol, TG and HDL-C) were measured via colorimetric method on a chemistry analyzer (AU5800 Analyzer, Beckman Coulter, Brea, CA). HbA1c was measured by HPLC method using Bio-Rad Variant II™ System (Bio-Rad Laboratories, Hercules, CA) in red blood cells.

### Statistical analysis

Study participants were divided into tertiles and the lowest tertile served as the reference group. We used conditional logistic regression models to compute the OR and corresponding 95% CI between adiponectin and T2D with adjustment for age (continuous), education level (primary school and below, secondary or above), smoking status (never, ever smoker), alcohol consumption (never, ever drinker), weekly moderate-to-vigorous physical activity levels (<0.5, ≥0.5 hours/week), history of hypertension (yes, no), BMI (continuous), and tertiles of biomarkers, including the ratio of TG to HDL-C, hs-CRP, random glucose and random insulin levels. We also calculated the T2D risk associated with per one log mg/L increment in adiponectin levels, in order to compare with previous studies^5^. To examine the impact of potential selection bias, we examined the adiponectin-T2D association among cases with HbA1c <6.5% or <6.0% and their matched controls. We stratified the analysis by sex, BMI, physical activity, smoking habits and hs-CRP level using unconditional logistical regression models with additional adjustment for sex and dialect group, and we used restricted cubic spline regression with 3 knots at 25^th^, 50^th^ and 75^th^ percentiles of adiponectin concentrations to examine the linearity of adiponectin-T2D association.

The predictive utility of adiponectin was examined by establishing three logistic regression models. Base model 1 included education level, physical activity, history of hypertension, BMI, TG, HDL-C, and hs-CRP; base model 2 additionally included random glucose and insulin. Since previous studies have shown that HbA1c levels outperform glucose levels in predicting T2D^44,45^, we further established base model 3 to substitute random glucose from base model 2 with HbA1c levels. The improvement in discrimination was examined by comparing AUC between each base model and the model plus plasma adiponectin levels^46^. Moreover, due to the limitation of AUC such as its insensitivity to model improvement^47^, we also evaluated the category-free NRI and IDI^48^. The goodness-of-fit of all models were assessed by Akaike information criteria (AIC), where lower AICs indicate better model fit.

### Meta-analysis

We performed an updated systematic review and meta-analysis of studies evaluating the association between adiponectin and T2D in adult populations since the date of the previous meta-analysis^5^. We followed the guidelines in Preferred Reporting Items for Systematic Reviews and Meta-Analyses and the Meta-analysis of Observational Studies in Epidemiology^49^. We searched systematically on PubMed for prospective human studies (including cohort, case-cohort, and nested case-control studies) assessing adiponectin-T2D associations between April 10, 2009 (the last date of literature research in the previous meta-analysis^5^) up to September 10, 2016. Our searches included both MeSH terms and key words without restrictions on language (Supplemental Material).

Studies were excluded if they were: (1) irrelevant (not using adiponectin as the exposure or T2D as the outcome); (2) review, editorial, commentary, meta-analysis, animal or experimental study; (3) not prospective study (case-control, cross-sectional, and genetic studies). Identified studies were screened for titles and abstracts first, and then potentially relevant articles were reviewed for full-text (Supplementary Figure S2). Studies included in the previous meta-analysis^5^ were also reviewed for full-text and added into the current meta-analysis. Data extraction was conducted independently by 2 authors (Y.W., R.M.). Extracted information included study characteristics (title, authors, publication year, study name, study design, follow-up length), participant characteristics (location, ethnic origin, sample size, number of incident T2D cases, gender composition, mean age or age range), adiponectin assay, T2D assessment, and analysis strategy (statistical models and covariates controlled). In case of multiple studies involving the same cohort, the most updated or relevant study was selected. We extracted relative risk estimates from the most fully-adjusted multivariable models. Studies qualities were assessed by the Newcastle-Ottawa Quality Assessment Scale^50^.

For the meta-analysis, RRs were used as the common measure of association, and ORs or hazard ratios (HRs) were considered as equivalent to RRs because of the low incident rate in most studies. To enhance consistency and improve comparability between studies, we transformed the originally reported RRs (per unit, per SD, per log increment, quartiles or quintiles) to a uniform comparison involving extreme tertiles of adiponectin levels using standard statistical methods which were described previously^51,52^. When RRs were reported in subgroups rather than the total sample in a study, a within-study RR was attained first using a fixed-effect analysis. RRs and corresponding 95% CIs were pooled by the DerSimonian-Laird random-effects models^53^, and fixed-effect models were used in sensitivity analyses. Between-study heterogeneity was evaluated by Cochran χ^2^ and *I* ^*2*^ Statistic^54^. Meta-regression analyses were used to examine the influence of certain factors on observed associations, including location, ethnicity, age, sex, follow-up duration, number of T2D cases, adiponectin assay, ascertainment of T2D, level of adjustment, and study quality. Stratified analyses were performed to evaluate the influences of baseline participant characteristics on the results. Potential for publication bias was examined by Egger’s test^55^. To detect any nonlinear association, restricted cubic spline regression model with three knots at 25%, 50% and 75% percentiles adiponectin distribution was applied. When the nonlinear hypothesis was rejected, a two-stage, log-linear, dose-response regression function was then used for both the fixed-effect and random-effect models to estimate the risk of T2D per 5 µg/mL increment in adiponectin levels^56^. Studies which reported case number, total number of participants, as well as adiponectin median value in each category were included in the dose-response meta-analysis. All *P* values were two-sided, and data were analyzed with STATA version 14 (Stata Corp, College Station, Texas).

### Data availability

The datasets generated during and/or analysed during the current study are available from the corresponding author on reasonable request.

## Conclusion

In conclusion, the strong, dose-dependent association between increased adiponectin levels and decreased T2D risk has been demonstrated in a Chinese population, and adiponectin may be a useful marker for T2D prediction among Chinese. Our meta-analysis strengthens the evidence that adiponectin is involved in T2D pathogenesis, and further clinical studies are needed to investigate the feasibility of targeting adiponectin through pharmacological, dietary and physical activity interventions to reduce the risk of T2D in high-risk population.

## Electronic supplementary material

Supplemental material
