# Supplementary material for: Plasma adiponectin levels and type 2 diabetes risk: a nested case-control study in a Chinese population and an updated meta-analysis
Source: Sci Rep. 2018 Jan 10;8:406. doi: 10.1038/s41598-017-18709-9 (PMC5762808; doi:10.1038/s41598-017-18709-9)
Supplement: Supplementary file 1 — Supplemental material [file 41598_2017_18709_MOESM1_ESM.pdf]

Plasma adiponectin levels and type 2 diabetes risk: a nested case-control study in a Chinese population and an updated meta-analysis

Yeli Wang, Rui-Wei Meng, Setor K. Kunutsor, Rajiv Chowdhury, Jian-Min Yuan, Woon-Puay Koh, An Pan

Online Supplemental Material

The following materials are included in the Online Supplemental Material.

1. Search strategies in PubMed
2. Supplemental Tables S1-S6
3. Supplemental Figures S1-S2

Supplemental Table S1. Pearson correlation coefficients between adiponectin and age, body mass index, levels of lipids, high-sensitivity C-reactive protein, random glucose and insulin in control participants, the Singapore Chinese Health Study

Supplemental Table S2. Odds ratio (95% confidence interval) for risk of type 2 diabetes according to tertiles of adiponectin, stratified by body mass index, physical activity, smoking, and high-sensitivity C-reactive protein levels, using unconditional logistic regression model, the Singapore Chinese Health Study

Supplemental Table S3. Summary statistics to assess adiponectin in predicting type 2 diabetes, the Singapore Chinese Health Study

Supplemental Table S4. Reclassification of type 2 diabetes cases and controls with no risk categories based on their plasma concentrations of adiponectin, the Singapore Chinese Health Study

Supplemental Table S5. Stratified analyses of adiponectin concentrations and risk of type 2 diabetes

Supplemental Table S6. The extracted data for the dose-response meta-analysis from 12 available studies

Supplemental Figure S1. Flowchart of the Singapore Chinese Health Study

Supplemental Figure S2. Flowchart of the meta-analysis

Search strategies in PubMed (September 10, 2016)

adiponectin [MeSH] OR adiponectin OR ADIPOQ OR ADPN) AND (diabetes mellitus [MeSH] OR diabetes OR diabetic OR diabetics)

Restricted to “human study”, and the time period was between April 10, 2009 to September 10, 2016.

Supplemental Table S1. Pearson correlation coefficients between adiponectin and age, body mass index, levels of lipids, high-sensitivity C-reactive protein, random glucose and insulin in control participants, the Singapore Chinese Health Study

Marker	Adiponectin	Age	BMI	TC	HDL-C	TG	Ratio of TG to HDL-C	Hs-CRP	Random glucose	Random insulin
Adiponectin	1.00									
Age	0.11[a]	1.00								
BMI	-0.15[a]	-0.01	1.00							
TC	0.04	-0.02	-0.02	1.00						
HDL-C	0.49[a]	-0.09[a]	-0.18[a]	0.29[a]	1.00					
TG	-0.43[a]	0.05	0.16[a]	0.13[a]	-0.54[a]	1.00				
Ratio of TG to HDL-C	-0.50[a]	0.07	0.19[a]	-0.01	-0.78[a]	0.95[a]	1.00			
Hs-CRP	-0.19[a]	0.06	0.26[a]	0.05[a]	-0.14[a]	0.11[a]	0.14[a]	1.00		
Random glucose	-0.01	0.08	0.03	-0.07	-0.06	0.06	0.07	0.04	1.00	
Random insulin	-0.20[a]	0.04	0.23[a]	-0.03	-0.29[a]	0.35[a]	0.36[a]	0.15[a]	0.66[a]	1.00

Abbreviations: BMI, body mass index; TC, total cholesterol; HDL-C, HDL cholesterol; TG, triglycerides; hs-CRP, high-sensitivity C-reactive protein.

[a]Correlation coefficients are statistically significant at *P* <0.05.

Supplemental Table S2. Odds ratio (95% confidence interval) for risk of type 2 diabetes according to tertiles of adiponectin, stratified by body mass index, physical activity, smoking, and high-sensitivity C-reactive protein levels, using unconditional logistic regression model, the Singapore Chinese Health Study[a]

Variables	Tertiles of adiponectin concentration			*P* for trend[b]	*P* for interaction[c]
	T1	T2	T3		
Gender					
Male	1.00	0.71 (0.38-1.31)	0.48 (0.18-1.25)	0.097	0.26
Female	1.00	0.67 (0.33-1.35)	0.21 (0.08-0.51)	0.001	
Body mass index					
<23.0 kg/m^2	1.00	1.09 (0.62-1.93)	0.59 (0.29-1.21)	0.21	0.041
≥23.0 kg/m^2	1.00	0.76 (0.47-1.22)	0.36 (0.19-0.69)	0.003	
Physical activity					
<0.5 hour/week	1.00	0.96 (0.64-1.43)	0.54 (0.23-0.54)	0.042	0.10
≥0.5 hour/week	1.00	0.61 (0.28-1.36)	0.21 (0.07-0.68)	0.01	
Smoking					
Never	1.00	0.84 (0.55-1.29)	0.49 (0.29-0.83)	0.011	0.27
Ever	1.00	0.93 (0.46-1.86)	0.28 (0.09-0.85)	0.07	
High-sensitivity C-reactive protein levels[d]					
≤1.2 mg/L	1.00	0.85 (0.48-1.53)	0.58 (0.29-1.15)	0.13	0.64
>1.2 mg/L	1.00	0.87 (0.55-1.39)	0.34 (0.18-0.65)	0.004	

[a]Model was adjusted for age at blood taken (continuous), gender (male, female), dialect group (Cantonese, Hokkien), smoking (never, ever smoker), alcohol intake (never, ever drinker), weekly activity (<0.5, ≥0.5 hours/week), education level (primary school and below, secondary or above), history of hypertension (yes, no), fasting status (yes, no), body mass index (continuous), high-sensitivity C-reactive protein (mmol/L), the ratio of triglycerides to HDL cholesterol, random glucose (mmol/L) and random insulin (mIU/L) (all in tertiles) except for the respective stratification variable.

[b]Linear trend was tested by using the median level of each tertile of adiponectin level.

[c]Interaction was tested between stratified variable (dichotomized) and adiponectin (per log increment) in the models.

[d]Median cutoff for high-sensitivity C-reactive protein was calculated among controls and defined as 1.2 mg/L.

Supplemental Table S3. Summary statistics to assess adiponectin in predicting type 2 diabetes, the Singapore Chinese Health Study

Variable	Multivariable model			
	Discrimination (AUC [95% CI])	Calibration (AIC)	NRI	IDI
Base model 1[a]	0.74 (0.71-0.77)	595		
Base model 1[a] + adiponectin	0.76 (0.73-0.79)[d]	566	33%	0.03
Base model 2[b]	0.82 (0.80-0.85)	389		
Base model 2[b] + adiponectin	0.83 (0.80-0.85)	376	19%	0.01
Base model 3[c]	0.87 (0.84-0.89)	329		
Base model 3[c] + adiponectin	0.87 (0.85-0.89)	325	12%	0.01

Abbreviations: AUC, area under the receiver operating characteristic curve; CI, confidence interval; AIC, Akaike information criterion; NRI, net reclassification improvement; IDI, integrated discrimination improvement.

[a]Base model 1 included education level (no, primary school, secondary and above), weekly moderate-to-vigorous activity (<0.5, 0.5-3.9 hours/week, ≥4 hours/week), history of hypertension (yes, no), body mass index (continuous), and tertiles of triglycerides, HDL cholesterol and high-sensitivity C-reactive protein.

[b]Base model 2 included variables in base model 1 plus tertiles of random glucose and random insulin.

[c]Base model 3 included variables in base model 1 plus tertiles of HbA1c and random insulin.

[d]Compared with the base model, the increment in AUC value was statistically significant (P <0.05).

Supplemental Table S4. Reclassification of type 2 diabetes cases and controls with no risk categories based on their plasma concentrations of adiponectin, the Singapore Chinese Health Study

Base model 1[a] + adiponectin	All	Assigned to higher diabetes risk	Assigned to lower diabetes risk	NRI	
Expected number of event participants	571	352	219	Among event participants	23.3%
Expected number of non-event participants	571	257	314	Among non-event participants	10.0%
				Overall original (95% CI)	33.3% (21.7%, 44.9%)
Base model 2[b] + adiponectin	All	Assigned to higher diabetes risk	Assigned to lower diabetes risk	NRI	
Expected number of event participants	571	334	237	Among event participants	17.1%
Expected number of non-event participants	571	280	291	Among non-event participants	2.0%
				Overall original (95% CI)	19.1% (6.8%, 31.4%)
Base model 3[c] + adiponectin	All	Assigned to higher diabetes risk	Assigned to lower diabetes risk	NRI	
Expected number of event participants	571	319	252	Among event participants	18.7%
Expected number of non-event participants	571	284	287	Among non-event participants	11.1%
				Overall original (95% CI)	12.3% (0%, 24.6%)

Abbreviations: NRI: net reclassification improvement.

[a]Base model 1 included education level (no, primary school, secondary and above), weekly moderate-to-vigorous activity (<0.5, 0.5-3.9 hours/week, ≥4 hours/week), history of hypertension (yes, no), body mass index (continuous), and tertiles of triglycerides, HDL cholesterol and high-sensitivity C-reactive protein.

[b]Base model 2 included variables in base model 1 plus tertiles of random glucose and random insulin.

[c]Base model 3 included variables in base model 1 plus tertiles of HbA1c and random insulin.

Supplemental Table S5. Stratified analyses of adiponectin concentrations and risk of type 2 diabetes

	Risk of type 2 diabetes			
	RR (95% CI)	*n* of prospective studies	I^2 (%)	*P* for Heterogeneity
Overall results	0.54 (0.47-0.61)	34	48.8	0.001
Trim and fill method	0.63 (0.50-0.76)	34	64.0	0.001
Sex				
Men	0.67 (0.50-0.90)	4	0	0.60
Women	0.32 (0.20-0.52)	2	0	0.47
Mixed	0.53 (0.45-0.62)	28	53.6	0.001
Study Location				
US	0.62 (0.49-0.79)	9	40.5	0.10
Europe or Oceania	0.50 (0.43-0.58)	18	29.6	0.12
Asia	0.48 (0.32-0.73)	7	69.4	0.003
Ethnicity				
Caucasian Whites	0.49 (0.43-0.57)	19	25.6	0.15
East Asians	0.51 (0.36-0.73)	8	64.3	0.01
Others, mixed	0.64 (0.48-0.85)	7	51.0	0.06
Mean age groups				
<60 years	0.53 (0.45-0.61)	24	49.8	0.003
≥60 years	0.56 (0.41-0.75)	10	47.1	0.049
Follow-up years				
<3 years	0.33 (0.06-0.75)	2	82.7	0.02
≥3 years	0.53 (0.47-0.61)	32	46.7	0.002
Sample size				
<2000	0.50 (0.42-0.59)	22	41.3	0.02
≥2000	0.53 (0.47-0.61)	12	60.2	0.004
Publication year				
<2010	0.57 (0.45-0.71)	14	43.6	0.04
≥2010	0.52 (0.44-0.61)	20	51.5	0.001
Laboratory assays for adiponectin				
ELISA	0.53 (0.45-0.63)	20	56.2	0.001
RIA	0.47 (0.29-0.75)	5	60.2	0.04
Others	0.60 (0.47-0.75)	9	13.6	0.32

Study quality[b]				
High (8-9)	0.56 (0.48-0.66)	24	44.1	0.01
Low (≤7)	0.47 (0.35-0.62)	10	62.2	0.01
Level of adjustment[c]				
Level 1	0.40 (0.34-0.47)	23	69.1	<0.001
Level 2	0.39 (0.30-0.50)	9	55.3	0.02
Level 3	0.53 (0.48-0.60)	28	22.9	0.14
Level 4	0.63 (0.49-0.80)	9	45.6	0.07

Abbreviations: RR, relative risk.

[a]The results are presented using the random-effects model.

[b]The study quality scored from 0 to 9, with higher score indicating higher quality.

[c]Level of adjustment was defined as below: level 1 was basic models without adjustment for metabolic biomarkers; level 2 was models adjusted for lipids and/or inflammatory markers; level 3 was models adjusted for either insulin sensitivity markers or glycaemia markers; level 4 was models adjusted for both insulin sensitivity markers and glycaemia markers.

Supplemental Table S6. The extracted data for the dose-response meta-analysis from 12 available studies

First author (reference)	Adiponectin category	Adiponectin median, μg/ml	Number of participants	Number of cases	RR (95% CI)
Keonig[1]	Tertile 1	3.81	298	57	1
	Tertile 2	6.32	294	31	0.63 (0.40-0.99)
	Tertile 3	10.6	295	27	0.81 (0.50-1.33)
Snijder[2] (male)	Quartile 1	8.3	145	18	1
	Quartile 2	9.8	151	16	0.79 (0.38-1.67)
	Quartile 3	10.6	143	14	0.71 (0.33-1.55)
	Quartile 4	12.4	145	12	0.61 (0.27-1.40)
Snijder[2] (female)	Quartile 1	3.5	170	27	1
	Quartile 2	7.2	170	14	0.58 (0.28-1.20)
	Quartile 3	8.2	170	11	0.47 (0.22-1.01)
	Quartile 4	15.8	170	6	0.27 (0.10-0.73)
Wannamathee[3]	Tertile 1	2.7	1189	55	1
	Tertile 2	5.4	1189	32	0.66 (0.42-1.04)
	Tertile 3	9.7	1189	18	0.40 (0.23-0.70)
Heidermann[4]	Quintile 1	8.1	775	548	1
	Quintile 2	13.9	449	221	0.46 (0.35-0.60)
	Quintile 3	17.9	366	139	0.31 (0.23-0.42)
	Quintile 4	21.7	300	73	0.21 (0.15-0.30)
	Quintile 5	28.4	284	57	0.17 (0.12-0.25)
Thorand[5]	Tertile 1	8.3	709	236	1
	Tertile 2	11.5	646	148	0.53 (0.37-0.76)
	Tertile 3	15.7	579	76	0.38 (0.27-0.53)
Kizer[6a]	Quartile 1	7.2	950	146	1

	Quartile 2	10.8	950	73	0.74 (0.55-1.01)
	Quartile 3	15.0	951	49	0.66 (0.46-0.96)
	Quartile 4	23.6	951	41	0.79 (0.50-1.23)
Li[7]	Quintile 1	4.3	964	86	1
	Quintile 2	6.3	697	33	0.58 (0.39-0.87)
	Quintile 3	7.9	487	15	0.41 (0.24-0.72)
	Quintile 4	10	431	10	0.34 (0.18-0.68)
	Quintile 5	13.9	429	20	0.72 (0.42-1.25)
Lilja[8] (male)	Quartile 1	3.1	388	195	1
	Quartile 2	7.5	294	101	0.83 (0.62-1.12)
	Quartile 3	10.5	224	31	0.40 (0.25-0.64)
	Quartile 4	15.2	235	43	0.52 (0.34-0.81)
Lilja[8] (female)	Quartile 1	4.6	346	149	1
	Quartile 2	11.6	274	76	0.70 (0.50-0.98)
	Quartile 3	16.2	218	21	0.38 (0.22-0.64)
	Quartile 4	23	218	21	0.44 (0.25-0.76)
Yamamoto[9]	Quartile 1	2.6	1145	76	1
	Quartile 2	6	1138	60	0.79 (0.55-1.12)
	Quartile 3	8.2	1174	47	0.60 (0.41-0.88)
	Quartile 4	11.8	1134	31	0.40 (0.25-0.64)
Marques-Vidal[10]	Quartile 1	3.7	913	71	1
	Quartile 2	7.3	969	58	0.97 (0.64-1.47)
	Quartile 3	11.3	969	48	0.84 (0.55-1.30)
	Quartile 4	17.3	991	31	0.64 (0.40-1.03)
Neville[11a]	Tertile 1	1.9	467	73	1
	Tertile 2	5.2	467	51	0.92 (0.60-1.43)

	Tertile 3	8.6	463	27	0.54 (0.29-0.99)
Current study	Quartile 1	5.3	420	275	1
	Quartile 2	7.5	305	161	0.65 (0.37-1.15)
	Quartile 3	9.4	234	91	0.62 (0.34-1.14)
	Quartile 4	12.8	183	44	0.33 (0.15-0.73)

Abbreviations: RR, relative risk.

[a]Numbers of cases were estimated from the incidence rates for type 2 diabetes reported in the studies.

Supplemental Figure S1. Flowchart of the Singapore Chinese Health Study

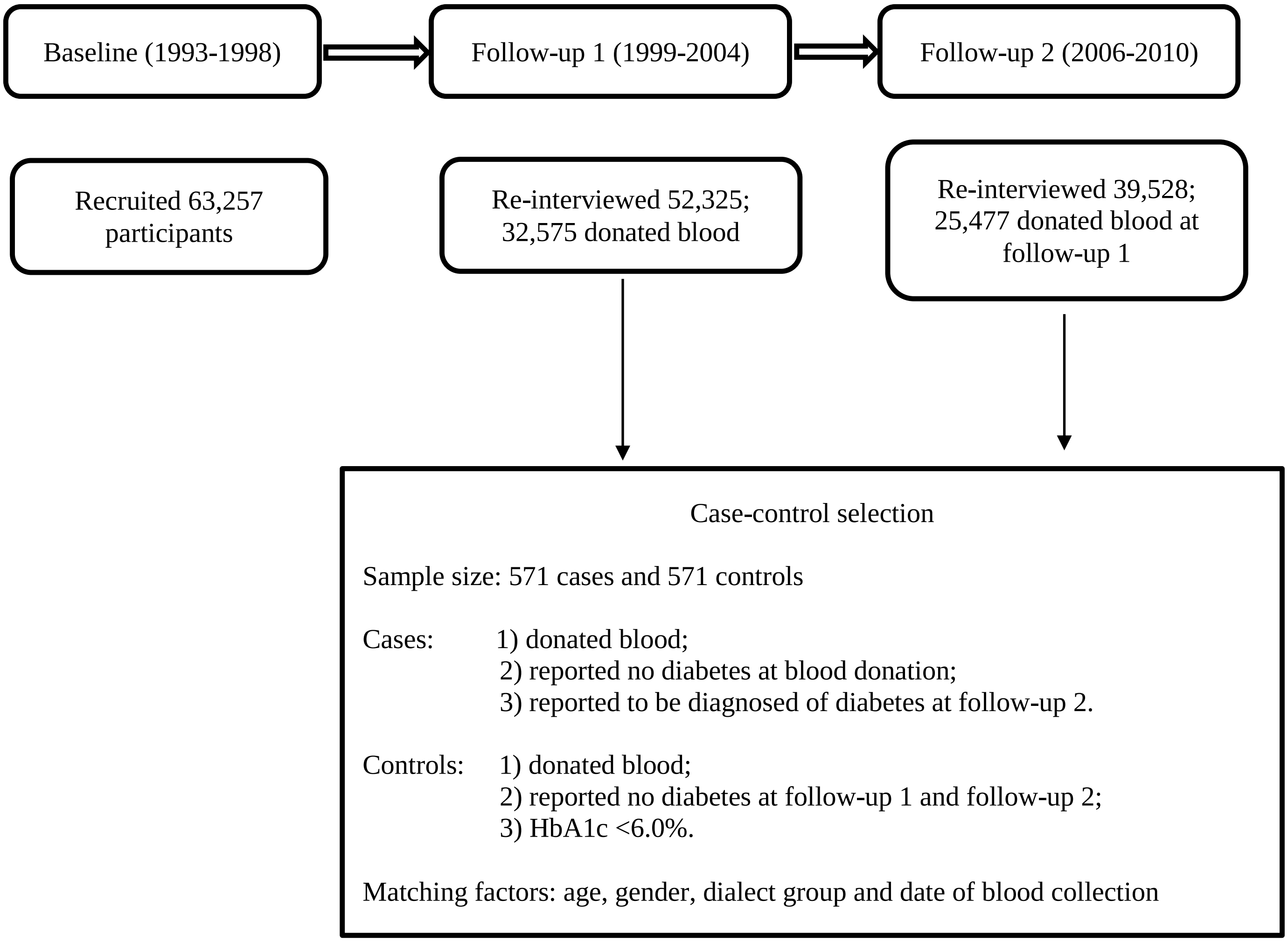

Supplemental Figure S2. Flowchart of the meta-analysis

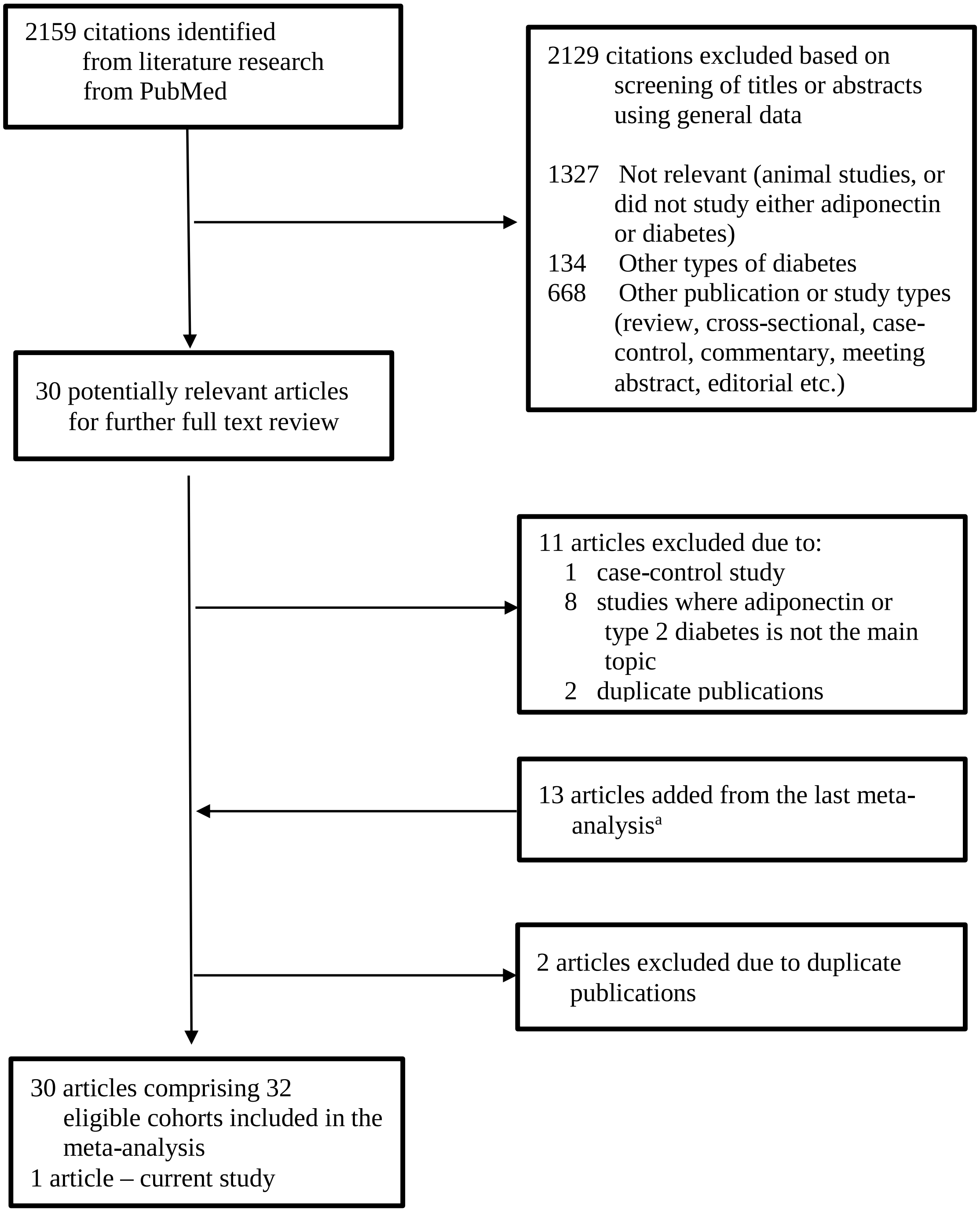

Figure legend

[a]The last meta-analysis referred to Li et al.

Li, S., Shin, H. J., Ding, E. L. & van Dam, R. M. Adiponectin levels and risk of type 2 diabetes: a systematic review and meta-analysis. *JAMA*. **302**, 179-188 (2009).

REFERENCES

1 Koenig, W., Khuseyinova, N., Baumert, J., Meisinger, C. & Lowel, H. Serum concentrations of adiponectin and risk of type 2 diabetes mellitus and coronary heart disease in apparently healthy middle-aged men: results from the 18-year follow-up of a large cohort from southern Germany. *J Am Coll Cardiol*. **48**, 1369-1377 (2006).

2 Snijder, M. B. *et al.* Associations of adiponectin levels with incident impaired glucose metabolism and type 2 diabetes in older men and women: the hoorn study. *Diabetes Care*. **29**, 2498-2503 (2006).

3 Wannamethee, S. G. *et al.* Adipokines and risk of type 2 diabetes in older men. *Diabetes Care*. **30**, 1200-1205 (2007).

4 Heidemann, C. *et al.* Total and high-molecular-weight adiponectin and resistin in relation to the risk for type 2 diabetes in women. *Ann Intern Med*. **149**, 307-316 (2008).

5 Thorand, B. *et al.* Associations between leptin and the leptin / adiponectin ratio and incident Type 2 diabetes in middle-aged men and women: results from the MONICA / KORA Augsburg study 1984-2002. *Diabet Med*. **27**, 1004-1011 (2010).

6 Kizer, J. R. *et al.* Total and high-molecular-weight adiponectin and risk of incident diabetes in older people. *Diabetes Care*. **35**, 415-423 (2012).

7 Li, Y., Yatsuya, H., Iso, H., Toyoshima, H. & Tamakoshi, K. Inverse relationship of serum adiponectin concentration with type 2 diabetes mellitus incidence in middle-aged Japanese workers: six-year follow-up. *Diabetes Metab Res Rev*. **28**, 349-356 (2012).

8 Lilja, M., Rolandsson, O., Norberg, M. & Soderberg, S. The impact of leptin and adiponectin on incident type 2 diabetes is modified by sex and insulin resistance. *Metab Syndr Relat Disord*. **10**, 143-151 (2012).

9 Yamamoto, S. *et al.* Circulating adiponectin levels and risk of type 2 diabetes in the Japanese. *Nutr Diabetes*. **4**, e130 (2014).

10 Marques-Vidal, P. *et al.* Adipocytokines, hepatic and inflammatory biomarkers and incidence of type 2 diabetes. the CoLaus study. *PLoS One*. **7**, e51768 (2012).

11 Neville, C. E. *et al.* The relationship between adipokines and the onset of type 2 diabetes in middle-aged men: The PRIME study. *Diabetes Res Clin Pract*. **120**, 24-30 (2016).
